# Physicians’ knowledge, attitude and perceptions towards vaccine-hesitant parents: a cross-sectional study

**DOI:** 10.1186/s12909-023-04590-w

**Published:** 2023-09-04

**Authors:** Asma R. Albaker, Samy A. Azer, Muneera AlKhorayef, Njoud K. Bin Dakheel, Shahad AlMutairi, Sarah AlHelal, Roaa Aljohani, Sarah Maghrabi

**Affiliations:** 1 Department of Paediatrics, College of Medicine, King Saud University, Riyadh, Saudi Arabia; 2 College of Medicine, Department of Medical Education, King Saud University, P O Box 2925, 11461 Riyadh, Saudi Arabia; 3 King Saud University, College of Medicine, Riyadh, Saudi Arabia

**Keywords:** Physicians, Children, Immunization, Vaccination, Vaccine-hesitant parents, Patient education

## Abstract

**Objectives:**

Research has shown that physicians are encountering an increase in vaccine-hesitant parents (VHPs) numbers. This study examined physicians' vaccination knowledge, vaccine-related discussions with VHPs, beliefs about and responses to vaccine hesitancy, and challenges faced while discussing immunization with VHPs.

**Methods:**

This cross-sectional, descriptive study was performed at King Saud University Medical City (KSUMC), Riyadh, Saudi Arabia, in September 2020. The data were collected through a questionnaire distributed via email. The sample comprised 90 physicians who routinely treat children and reported they frequently have appropriate vaccine discussions when encountering VHPs.

**Results:**

Ninety participants (59% were females) completed the questionnaire. Of these, 37.8% were from family medicine, 7.8% from primary care, and 54.4% from paediatrics. The most discussed topics were vaccine necessity, reasons for vaccine refusal, and vaccine safety. Seventeen participants (18.8%) reported being extremely confident, and (42.2%) were confident in their vaccine-specific knowledge. Regarding confidence in communication skills, 22.2% reported being extremely confident and (45.6%) were confident. Determinants of higher confidence in the knowledge and communication skills were physician age (*p* = 0.001 and *p* = 0.0001, respectively), years of practice (*p* = 0.002 and (*p* = 0.005), and patients seen per workday (*p* = 0.0001 and *p* = 0.024). Other factors such as physician sex (*p* = 0.062), the field of practice (*p* = 0.329), and hours of work per week (*p* = 0.061) were not significantly different. Forty-six (51%) physicians sometimes find it challenging to conduct appropriate vaccine-related discussions because of having too many other issues to discuss during the consultation. Furthermore, 53 (59%) participants agreed/strongly agreed that parental refusal to vaccinate would raise suspicions of negligence. On the other hand, 59 (65%) disagreed/strongly disagreed that parental refusal of vaccines is a parental right. Participants expressed the need to refer VHPs to a specialised advisory clinic with excellent experience and negotiation skills to overcome the challenges.

**Conclusion:**

Vaccine safety and necessity are the topics of most concern to VHPs, and a knowledgeable physician with competent communication skills is critical in responding to such situations. This study highlights the most reported barriers to successful vaccine-related discussions. It raises underlying ethical principles such as parental autonomy and the need to train physicians in VHPs. To train physians for succucful vaccine counceling of VHPs.

**Supplementary Information:**

The online version contains supplementary material available at 10.1186/s12909-023-04590-w.

## Introduction

Immunization is a well-recognized public health measure for reducing the mortality and morbidity of serious communicable diseases [1]. According to the WHO’s State of the World’s Vaccines and Immunization, if countries could raise global vaccine coverage to a rate of 90%, 2 million deaths a year in children under five years of age could be prevented [2]. However, some parents exhibit vaccine hesitancy, which challenges the medical community and has a detrimental effect on vaccination rates and herd immunity [3]. “Vaccine-hesitant parents” (VHPs) term is defined in the literature as parents who delay or refuse the vaccination of their children despite the availability of vaccines [3, 4]. Several reasons behind the increase in vaccine hesitancy among parents include misinformation about vaccines and their safety, distrust in the healthcare system, and the influence of anti-vaccination reports in social networks [3, 5, 6]. Some parents might view immunization as unnecessary because the benefits are not visible and direct to them.

In contrast**,** as highlighted in social media, possible risks could contribute to parents’ worries, hesitancy, or refusal to vaccinate their children. Furthermore, the diseases that the vaccines prevent are often unknown to the general population or becoming rare in the community, such as poliomyelitis and diphtheria [3, 7, 8]. Others may refuse specific vaccines, such as the MMR vaccine, because they believe it can cause neurodevelopmental delay or autism, or because they are concerned about serious side effects [3, 6, 9].

Healthcare providers are assumed to be the primary and reliable**,** up-to-date source of information for parents about child immunization; thus, they play a central role in parents’ vaccination decision-making processes [3, 9]. Paediatricians**,** primary care, and family medicine physicians are expected to provide appropriate informative messages to parents to achieve high immunization rates [4, 10]. However, several barriers can limit relevant discussions of physicians with VHPs. In two studies in Australia, the barriers paediatricians or primary care physicians reported were a lack of time for consultations and a lack of confidence in answering patients’ questions [11, 12]. One showed that 15% of paediatricians skipped discussing vaccine-related issues with parents [11]. Moreover, A study found 21% of paediatricians and 4% of family medicine physicians dismiss a child from their practice if the parent refuses one or more vaccines [13]. Furthermore, a study from Italy reported that only 42.3% of surveyed paediatricians knew all the recommended vaccines for infants, with higher percentages of female doctors, doctors who worked more hours per week, and doctors who used immunization guidelines in practice possessing such knowledge [14].

In addition to barriers to health care providers’ counselling of VHPs, physicians have the opportunity for direct encounters with VHPs, which may negatively impact physicians due to parents’ resistance toward vaccination. A study showed that half of the paediatricians who regularly encountered VHPs suffered higher levels of burnout and lower levels of job satisfaction than those who did not periodically encounter VHPs [12]. Furthermore, another study reported that physicians experienced interaction with VHPs to be a direct challenge to their identities as trusted and reliable authority figures [12]. Research has shown that communication between healthcare providers and VHPs is important [4, 10, 15]. For example, a Cochrane review showed that parents’ willingness to vaccinate their children improved when healthcare providers used face-to-face education rather than providing materials or online resources [16]. Therefore, effective communication practices with VHPs can help change their views about vaccination. Consequently, training and educational intervention in this vaccine-related knowledge is required to ensure that healthcare providers have the appropriate knowledge and communication skills [4, 15].

Considering these issues, this study aimed to assess physicians’ attitudes and perceptions regarding the importance of discussing vaccines with VHPs, determine their strategies for doing so, and identify the challenges physicians face when they encounter VHPs.

## Methods

### Study design

In Saudi Arabia, vaccinations are provided by paediatrician, family medicine or primary care practices. This observational, cross-sectional, questionnaire-based study was conducted at King Saudi University Medical City (KSUMC) and its affiliated hospitals in Riyadh, Saudi Arabia, between September 2020 and March 2021. The target population was physicians directly involved in childhood vaccination administration, including paediatricians, family medicine, primary care physicians, residency trainees in these specialities at KSUMC, residents, fellows, consultants, and. We sent an invitation and the questionnaire to all 220 physicians from the departments of paediatrics and primary care and family medicine in KSUMC. The study was approved by the Institutional Review Board (IRB) of King Saudi University (No. E-20–5447) in November 2020. A written consent was obtained before the start of the questionnaire. Participation was voluntary, and all responses remained anonymous.

### The questionnaire

We extrapolated questionnaire dimensions and questions from published articles and literature review [9, 11–14, 17, 18]. We modified the themes extracted from previous studies to fit the study purpose and the Saudi medical community. The questionnaire was in English which is the language of medical academic community in Saudi Arabia.

Few dimensions in relation to vaccine-related ethics and professional identity that appeared to be rarely been addressed in a questionnaire format before were drawn from a qualitative study and ethic review studies [12, 19–21]. The questionnaire was divided into six parts. Part A covered demographics, Part B covered experience with incompletely vaccinated children, Part C covered knowledge, beliefs, and opinions regarding vaccination. Part D covered dealing with vaccine-hesitant parents, Part E covered responses to refusal to vaccinate, and Part F covered challenges and strategies (Appendix).

### Questionnaire distribution

The next step was to use Google Forms to create an electronic questionnaire version**. **The invitations to participate and the questionnaire were sent to participants’ e-mail addresses with the help of the departmental staff database and their cell phones via a phone messaging system—WhatsApp or Short Messaging System (SMS).

### Pilot study

The questionnaire was pilot tested with ten physicians in their internship who were not part of our intended sample. The purpose of the pilot study was to evaluate each question's content, face validity, readability, and clarity. Based on the written feedback received during the pilot test**, **the questionnaire was modified before being used in the study. For example, we added "general practitioner" to the response options relating to the level of expertise.

### Data analysis

The data were analysed using SPSS version 24.0 statistical software. Descriptive statistics (frequencies, percentages, means and standard deviations) are used to describe the variables. Bivariate statistical analysis was carried out using the Student’s t-test and one-way analysis of variance to compare the mean knowledge, challenge scores, attitude and opinions of physicians toward VHPs concerning the sociodemographic variables that had two or more categories**.** A *p*-value of < 0.05 was considered significant.

## Results

### Participants' demographics

Table 1 shows that more than half of the participants were females. Most were from peaediatrics (54.4%), followed by family medicine (37.8%), and then primary care(7.8%). (71.1%) of the participants had less than ten years of experience, while (6.7%) of them had more than 30 years of experience. Only (12.2%) saw more than 25 patients per day, and (40%) saw ten or fewer patients per day.

**Table 1:** Participants’ demographics

Sex Number (%)		Age group		Level of expertise		Field of practice	
**Female**	53 (59)	**25–34**	59 (65.5)	**General practitioner**	11 (12.2)	**Primary care**	7 (7.8)
**Male**	37 (41)	**35–44**	15 (16.7)	**Year 1 or 2 resident**	25 (27.8)	**Family medicine**	34 (37.8)
		**45–54**	7 (7.8)	**Years 3, 4 or 5 resident**	17 (18.9)	**Paediatric medicine**	49 (54.4)
		**55–64**	7 (7.8)	**Specialist**	5 (5.6)		
		** > 64**	2 (2.2)	**Fellow**	9 (10)		
				**Consultant**	23 (25.6)		
**Experience Years**		**Patients seen per day**				**Workload hrs per week**	
**10 years or fewer**	64 (71.1)	**10 patients or fewer**		36 (40)		**30 h or fewer**	24 (26.7)
**11–20**	14 (15.6)	**11–15**		26 (28.9)		**31–50**	51 (56.7)
**21–30**	6 (6.7)	**16–20**		13 (14.4)		** > 50**	15 (16.7)
** > 30**	6 (6.7)	**21–25**		4 (4.4)			
		** > 25**		11 (12.2)			

### Participants' confidence in their vaccine-related knowledge and communication skills

The participants' confidence level in knowledge and communication skills when discussing vaccine-related issues with parents showed that seventeen (18.8%) of the participants reported that they were "extremely confident", and 42.2% were "confident", while only 4.4% were "not confident". Regarding their communication skills with VHPs, 20 (22.2%) of the respondents were "extremely confident" and 45.6% were "confident", and only 3.3% were "not confident".

Comparing the mean score of confidence in vaccine-related knowledge with participants’ demographics showed that the higher the age (*p* < 0.001), number of years in practice (*p* = 0.002), and number of patients seen by the physician per day (*p* = 0.0001) were significantly associated with a higher confidence score in discussing vaccine-related matters. Other factors are shown in Table 2.

**Table 2:** Comparison of the mean scores of confidence in vaccine related knowledge against the socio-demographic characteristics

Characteristics		Level of confidence in vaccine-related knowledge					
		Mean (SD)	Not very confident N (%)	Somewhat confident N (%)	Confident N (%)	Extremely Confident N (%)	*p*-value
**Age group**	25–34	2.59 (0.81)	4 (100)	24 (77.4)	23 (60.5)	8 (47.1)	*p* < 0.001
	35–44	2.67 (0.62)	0 (0.0)	6 (19)	8 (21)	1 (5.9)	
	45–54	3.71 (0.48)	0 (0.0)	0 (0.0)	2 (5.3)	5 (29.4)	
	55–64	3.00 (0.57)	0 (0.0)	1 (3.2)	5 (13.2)	1 (5.9)	
	> 64	4.00 (0.00)	0 (0.0)	0 (0.0)	0 (0.0)	2 (11.8)	
**Sex**	Male	2.78 (0.82)	2 (50)	11 (35.5)	17 (44.2)	7 (41.2)	*p* = 0.785
	Female	2.74 (0.81)	2 (50)	20 (64.5)	21 (55.3)	10 (58.8)	
**Level of expertise**	General practitioner	2.18 (0.98)	1 (25.0)	3 (9.7)	4 (10.5)	3 (17.6)	*p* = 0.154
	Yrs 1 or 2 resident	2.72 (0.98)	2 (50.0)	10 (32.3)	6 (15.8)	7 (41.2)	
	Yrs 3,4,5 resident	2.35 (0.49)	0 (0.0)	11 (35.5)	6 (15.8)	0 (0.0)	
	Specialist	3.2 (0.84)	0 (0.0)	1 (3.2)	2 (5.3)	2 (11.8)	
	Fellow	2.67 (0.71)	1 (25.0)	1 (3.2)	7 (18.4)	0 (0.0)	
	Consultant	3.00 (0.67)	0 (0.0)	5 (16.1)	13 (34.2)	5 (29.4)	
**Field of practice**	Primary care	2.86 (1.07)	1 (25.0)	1 (3.2)	3 (7.9)	2 (11.8)	*p* = 0.119
	Family medicine	2.53 (0.86)	2 (50.0)	18 (58.1)	8 (21.1)	6 (35.3)	
	Paediatric medicine	2.76 (0.81)	1 (25.0)	12 (38.7)	27 (71.1)	9 (52.9)	
**Years of practice**	10 or fewer	2.56 (0.77)	4 (100.0)	27 (87.1)	26 (68.4)	7 (41.2)	*p* = 0.002
	11–20	3.07 (0.73)	0 (0.0)	3 (9.7)	7 (18.4)	4 (23.5)	
	21–30	3.50 (0.55)	0 (0.0)	0 (0.0)	3 (7.9)	3 (17.6)	
	> 30	3.33 (0.82)	0 (0.0)	1 (3.2)	2 (5.3)	3 (17.6)	
**Patient seen per workday**	10 or fewer	2.39 (0.69)	3 (75.0)	17 (54.8)	15 (39.5)	1 (5.9)	*P* = 0.0001
	11–15	2.73 (0.78)	1 (25.0)	9 (29.0)	12 (31.6)	4 (23.5)	
	16–20	3.15 (0.80)	0 (0.0)	3 (9.7)	5 (13.2)	5 (29.4)	
	21–25	3.00 (0.82)	0 (0.0)	1 (3.2)	2 (5.3)	1 (5.9)	
	> 25	3.45 (0.69)	0 (0.0)	1 (3.2)	4 (10.5)	6 (35.3)	
**Workload hours per week**	30 or fewer	2.625 (0.87)	1 (25.0)	12 (38.7)	6 (15.8)	5 (29.4)	*p* = 0.66
	31–50	2.80 (0.75)	2 (50.0)	14 (45.2)	27 (71.1)	8 (47.1)	
	> 50	2.8 (0.94)	1 (25.0)	5 (16.1)	5 (13.2)	4 (23.5)	

Comparing the mean confidence level in communication skills with participants’ demographics showed that the higher the age (*p* = 0.0001), level of expertise (*p* = 0.005), number of years in practice (*p* = 0.0001), number of patients seen per day by the physician (*p* = 0.024) were correlated with the highest reported confidence in communication skills in vaccine-related discussion. Other factors are shown in Table 3.

**Table 3:** Comparison of the mean scores of confidence level in communication with VHP against the socio-demographic characteristics

**Parameter**		**Mean out of 4** ** (SD)**	**Test statistics**	***p*-Value**
**Age group**	25–34	2.695 (0.7934)	F(4, 85) = 6.632	*p* = 0.0001
	35–44	2.667 (0.4880)		
	45–54	3.714 (0.4880)		
	55–64	3.571 (0.5345)		
	> 64	4 (0.0)		
**Sex**	Male	3.054 (0.7433)	T(88) = 1.893	*p* = 0.062
	Female	2.736 (0.8122)		
**Level of expertise**	General practitioner	2.909 (0.8312)	F(5, 84) = 3.655	*p* = 0.005
	Year 1 or 2 residents	2.840 (0.8981)		
	Year 3, 4 and 5 residents	2.235 (0.4372)		
	Specialist	3.2 (0.8367)		
	Fellow	3.111 (0.6009)		
	Consultant	3.174 (0.7168)		
**Field of practice**	Primary care	3 (1)	F(2, 87) = 1.126	*p* = 0.329
	Family medicine	2.706 (0.6291)		
	Paediatric medicine	2.959 (0.8650)		
**Years of practice**	10 or fewer	2.656 (0.7605)	F(3, 86) = 8.473	*p* = 0.0001
	11–20	3.071 (0.6157)		
	21–30	3.667 (0.5164)		
	> 30	3.833 (0.4082)		
**Patients seen per workday**	10 or fewer	2.639 (0.7232)	F(4, 85) = 2.955	*p* = 0.024
	11–15	2.962 (0.8709)		
	16–20	2.692 (0.8549)		
	21–25	3.250 (0.5)		
	> 25	3.455 (0.5222)		
**Work hours per week**	30 or fewer 31–50 > 50	2.542 (0.7790) 3 (0.7483) 2.933 (0.8837)	F(2, 87) = 2.885	*p* = 0.061

### Physicians' attitudes and opinions regarding vaccination

Twenty-seven (30%) of the respondents "strongly agreed" and 28.9% "agreed" with the statement that “refusal of vaccinations by parents for non-medical reasons should raise suspicions of negligence and questions about the child’s welfare”. Eighteen (20%) of the participants "strongly agreed" and 30% "agreed" with the statement that “refusal of vaccinations by parents for non-medical reasons should be dealt with as a child protection case and should be raised to the childhood welfare authority”. Fifty-nine (65.5%) of the respondents “Strongly disagree” and “disagreed” with the statement that "refusal of vaccines is a parental right". Responses to other related statements are shown in Table 4.

**Table 4:**
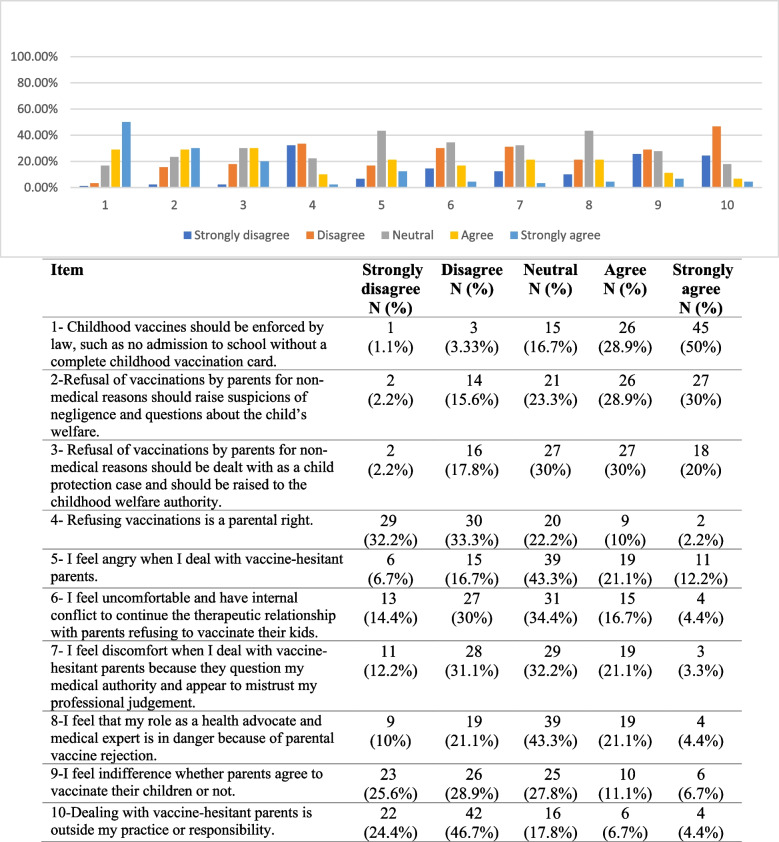
Physicians’ attitudes and opinions regarding vaccination

### Frequency of appropriate vaccine-related discussions

Among physicians only eleven (12.2%) reported that they discussed and explained vaccination with VHPs in every encounter. Participants reported that they always discuss topics about “vaccine necessity” (55.6%), “explore the reasons of vaccine refusal” (53.3%), and “vaccine safety” (53.3%), and “vaccine efficacy” (40%).

### Physician responses to refusal to vaccinate

Table 5 summarises the physicians’ responses and actions they would take upon parental refusal to have their children vaccinated. Fourteen (15.5%) always required parents to sign a form to maintain records about parents refusal. Ten (11.1%) always advise parents who have refused vaccination that they should inform urgent care physicians about the vaccination status of their children. Other responses/actions taken by physicians and their frequency are shown in Table 5.

**Table 5:** Physicians response to refusal to vaccinate

How often do you:	Rarely n (%)	Sometimes n (%)	Frequently n (%)	Always n (%)
Require parents to sign a form to maintain records that the parents refuse vaccinations for non-medical reasons	54 (60)	12 (13.33)	10 (11.11)	14 (15.55)
Advise parents who have refused vaccinations that they should inform on-call or urgent care physicians about their children’s vaccination statuses	30(33.33)	28 (31.11)	22 (24.44)	10 (11.11)
Dismiss families from your practice if they refuse one or more vaccines in the primary series	58 (64.44)	19 (21.11)	12 (13.33)	1 (1.11)
Schedule extra visits solely to address vaccination concerns	31 (34.44)	34 (37.77)	18 (20)	7 (7.77)
Advise parents who refuse certain vaccines that their children should wear MedicAlert tags or bracelets	55 (61.11)	14 (15.55)	16 (17.77)	5 (5.55)
Hold group information meetings or provide pamphlets to educate parents about vaccine-related information	53 (58.88)	17 (18.88)	10 (11.11)	10 (11.11)

### Challenges for performing vaccine discussions

The majority of physicians reported “lack of time allocated for vaccine talk” is “sometimes challenging”, 45 (50%), and is “always challenging”, 17 (18.9). Another challenge was “too many other issues to discuss during the clinic time” reported by 46 (51.1%) as “sometimes challenging”, and by 14 (15.5%) as “always challenging.” A third challenge was “lack of knowledge about specific vaccines” reported by 25 (27.8%) as “sometimes challenging”, and by 13 (14.4%) as “always challenging.”. Other challenges are shown in Table 6.

**Table 6:** Challenges for performing vaccine discussions

Challenging factors and their extent	Never been challenging n (%)	Rarely challenging n (%)	Sometimes challenging n (%)	Always challenging n (%)
Too many other issues to discuss	7 (7.8)	23 (25.6)	46 (51.1)	14 (15.5)
Lack of time	6 (6.7)	22 (24.4)	45 (50)	17 (18.9)
Concern about conflict or hostility in relationship	18 (20)	35 (38.9)	26 (28.9)	11 (12.2)
Lack of knowledge	16 (17.8)	36 (40)	25 (27.8)	13 (14.4)
Lack of knowledge about current national vaccination schedule	25 (27.8)	37 (41.1)	19 (21.1)	9 (10)
Too many recent changes to current national vaccination schedule	20 (22.2)	32 (35.6)	27 (30)	11 (12.2)
Lack of knowledge about how vaccines work	20 (22.2)	41 (45.6)	22 (24.4)	7 (7.8)
Lack of knowledge about vaccine-preventable diseases	31 (34.4)	34 (37.8)	18 (20)	7 (7.8)

**Fig. 1:**
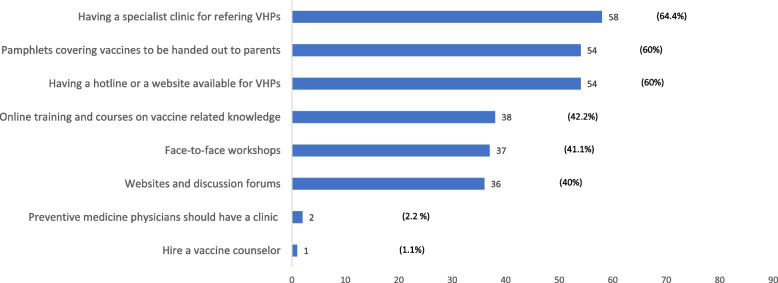
Strategies to overcome challenges faced by physicians handling vaccine-hesitant parents (VHPs)

The strategies and tools rated by participants as highly effective in aiding successful vaccine-related discussions are shown in Fig. 1. Of these, 58 (64.4%) welcomed having a specialised clinic to which they can refer VHPs, and 54 (60%) indicated the importance of having a readily available pamphlet and establishing a hotline or a website for VHPs to discuss vaccination issues. In addition, 38 (42.2%) indicated the need for online training modules, and the need for further training on child immunization.

## Discussion

In this study, we report on the attitudes, perceptions, and actions of physicians and trainees in paediatrics, family medicine, and primary care when faced with vaccine-hesitant parents (VHPs). Most participants in this study declared that they have confidence in vaccine-related knowledge but needed help with certain aspects of knowledge to deliver appropriate counselling when opposed by VHPs. A small proportion of participants reported feeling angry or stated that their identity as physicians had been shaken when dealing with VHPs and the parents' refusal of vaccination. On the other hand, around 50% of participants would take more stringent actions and reported that they would contact the children's welfare authority when dealing with parental refusal to vaccinate. However, this strict action is not supported by paediatric societies or legal authorities [4, 5].

This study also demonstrated that physicians’ age, years of practice, and the number of patients seen per day, were among the determinants of having higher confidence in their knowledge when discussing vaccine-related matters with parents. Also, the physician's confidence level in communication skills was associated with the physician's age, level of expertise, number of years in practice and the number of patients seen per day. Interestingly, an Italian study showed similar findings and found that being a female physician was associated with being more knowledgeable about vaccine-related issues [14]. However, our study showed no association between physician sex and knowledge level or communication skills related to vaccination discussions.

Attainment of physicians of a certain magnitude of vaccine-related knowledge and confidence is essential to counter the views and the hesitancy that may arise when conversing with and counselling VHPs [3].

Notably, physicians from family medicine, primary care, and paediatrics in Saudi Arabian hospitals are responsible for the immunization of children. Therefore, it is concerning that only 40% of the physicians in this study reported that they frequentlyhave appropriate vaccine-related conversations with VHPs. In contrast, one-third of physicians reported omitting these discussions altogether. A study from Australia reported a higher discussion rate of vaccines in every encounter with a VHP, with 66% of paediatricians always having such discussion [11]. The study by Freed et al. from the United States reported that paediatricians were more likely to provide additional vaccine information than family medicine physicians [9].

Regarding the topics discussed, this study shows that vaccine necessity and safety were the most frequent, followed by vaccine efficacy, catch-up schedules, and MMR vaccine concerns. This finding is consistent with studies conducted by paediatricians or family medicine physicians in the United States [9], Australia [11], and Italy [14]. From a parental perspective, Alsubaie et al. found that 53% of Saudi Arabian VHPs were concerned about vaccine safety and believed vaccines were unimportant or ineffective [6].

In Saudi Arabia, the national childhood vaccination schedule has been changed in the last five years. For example, adding the rotavirus vaccine and delaying the Bacillus Calmette-Guerin (BCG) vaccine to a 6-month jab instead of at birth [22]. Nearly half of the respondents in our study mentioned that these changes made it “always” or “sometimes” challenging to follow the vaccination schedule and know how vaccines work. One-third of physicians reported that their lack of knowledge about specific vaccines, how vaccine works, and vaccine-preventable diseases were burdensome and overwhelming in conducting proper counselling [23, 24].

Regardless of these challenges, physicians are obliged to educate parents about the benefits and risks of vaccines and their effects in protecting the community from serious infectious diseases [4, 15, 25]. They should also explain that immunization benefits both the child and public health and far outweigh any risks [21, 26]. The implications of vaccine refusal are measurable with the re-emergence of potentially life-threatening vaccine-preventable infectious diseases [7, 8].

In this study, around half of the physicians questioned the ethics of parental refusal to vaccinate their children and suggested that the refusal of vaccines was a sign of negligence or should be reported to the authorities. Moreover, 65% of the surveyed physicians did not believe parents have the right to refuse vaccination.

On the other hand, physicians with a higher level of expertise tended to agree that vaccine refusal is a parental right. It is crucial to note that there are very few legal proceedings to act against parental wishes regarding vaccination [19]. In the United States, a court review article showed that parental refusal to vaccinate in some cases may constitute neglect [19, 27]. How physicians should react to vaccine refusal is a discordant matter and has been discussed in a few publications from the American Academy of Paediatrics (AAP) [5, 25] and the Canadian Paediatric Society [4, 5]. In AAP and other reviews, most ethicists found no ethical background to vaccinate against parents' wishes; such an act breaches parental autonomy and creates a toxic atmosphere for continuing public health measures [5, 25, 27]. In Saudi Arabia, no policies support that physicians can make allegations of neglect based exclusively on refusal to vaccinate. To our knowledge, Saudi Arabia has no clear policy regarding this matter except the school-entry mandatory requirement to have a complete childhood vaccination card [28].

This study also investigated the physicians' actions in response to parental vaccine hesitancy. One-quarter to one-third of physicians reported that they took further measures to ensure their safety in dealing with unvaccinated children, for example, requiring parents to sign a vaccine-refusal document to add to the child's medical chart and asking parents to alert on-call emergency physicians of the child's vaccination status when they visit the emergency room. Also, ask parents to have the child wear a bracelet or MedAlert ID. Approximately 14% of the physicians in our study reported they would dismiss patients who refuse vaccination in our study, which is similar to the finding of an American Study that 21% of paediatricians and 4% of family medicine physicians would ignore patients if their parents refused to vaccinate their children [13]. Experts argue against dismissing patients because this will lead to lost opportunities to discuss and convince them. In contrast, others would justify the dismissal because vaccine refusal undermines the physician–parent relationship and unimmunized children will pose a risk of contracting and spreading vaccine-preventable infections to other vulnerable patients, such as immunocompromised infants who are not yet fully vaccinated [26]. Therefore, we should encourage physicians not to dismiss vaccine refusers from practice, use motivational interviewing techniques and clear language to present evidence for disease risk [4, 5, 25]. Physicians might advise the parent to seek medical care from another physician [4, 5, 25, 26].

The physicians identified the most significant barriers to conducting vaccine-related counselling were a lack of allocated time for such counselling and having many other issues to discuss with parents. Indeed, only one-quarter of the physicians said they would book an extra visit solely to discuss vaccinations.

The study also revealed that physicians in family medicine were more likely to sense that the therapeutic relationship and their status as medical experts were shaken by the parental refusal of vaccination than were paediatricians. A recent study from Kansas to explore the perceptions of family medicine physicians as to why parents in Kansas may be vaccine hesitant suggested that physicians must try and implement discussions or interventions suited to varying reasons why parents/guardians refuse vaccines in order to combat unwarranted concerns about vaccination [29].

Participants indicated that they need more resources to face these challenges in child vaccination and further training on discussing and addressing the concerns of VHPs. A significant proportion of physicians indicated that they would appreciate a specialised clinic to refer VHPs to a specialist with a higher level of expertise in this field. Others welcomed strategies to overcome barriers, including having readily available fact sheets and having a hotline number that VHPs could call to discuss vaccination issues and their concerns. Several studies showed that introducing an online module or face-to-face training for physician education in VHPs would support physicians and could ease their challenges [3, 5, 15].

Our study is not free from limitations. First, it was designed to be a cross-sectional study and only represents one state in the Kingdom of Saudi Arabia. Second, the study was based on a questionnaire, so it relied on the written responses and the possible limitations associated with questionnaire-based studies. Third, We have not addressed ethical issues against and for a patient's refusal to vaccinate their children or the professional basis for physician reaction to VHPs in this study. A further limitation was the relative response rate of only 41%, despite our effort to reach physicians by email and WhatsApp with three reminders. Factors such as COVID-19, the lockdown, and physicians' busy schedules might have compromised their ability to complete the questionnaire. These study limitations mean that our study results may not be generalizable to all physicians who interact with VHPs.

## Conclusions

Physicians from the department of family medicine, primary care, and paediatrics are essential in maintaining high vaccination rates in Saudi Arabia. Vaccine safety and necessity are the most significant concern to VHPs, and a knowledgeable physician is critical in responding to such situations. This study highlights the barriers to having a successful vaccine-related discussion, including the need for more time and having too many issues to discuss with parents. Further studies, preferably qualitative, should be undertaken to elaborate on the ethical argument against or for a parental right to refuse vaccination and on physicians' ethical and moral understanding of this issue. The study also highlights that further educational resources and training are crucial for successful vaccine counselling of VHPs.

### Supplementary Information

**Additional file 1.**

## Data Availability

The datasets used and analysed during this study are available from the corresponding author on reasonable request.

## Glossary

A primary care physician: is a specialist in family medicine who provides care to the undifferentiated patients at the point of first contact and takes continuing responsibilities for providing the patients with comprehensive care. The care may include preventive and acute care.
A family medicine: physician is involved in managing most health conditions in a community using holistic care and encompass patient-centered care across the family life-cycle
A pediatrician: is a physician concerned in the health and wellfare of children. They maybe involved in primary care pediatrics and pediatric medical subspecialities
A resident: is a physician who has completed the medical school and is receiving training in a specialized area such as surgery, internal medicine, radiology or others.
