# Supplementary material for: Physicians’ knowledge, attitude and perceptions towards vaccine-hesitant parents: a cross-sectional study
Source: BMC Med Educ. 2023 Sep 4;23:637. doi: 10.1186/s12909-023-04590-w (PMC10478439; doi:10.1186/s12909-023-04590-w)
Supplement: Supplementary file 1 — Additional file 1. [file 12909_2023_4590_MOESM1_ESM.docx]

**
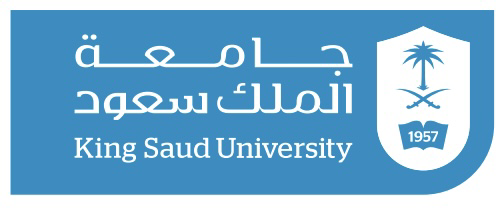
**

**Assessment of Knowledge, Attitudes and Perceptions of Physicians Toward Vaccine-Hesitant Parents**

Principal Investigator:

Asma Albaker, MD, FRCPC, Department of Paediatrics, College of Medicine, King Saud University.

Email: ASMA.ALBAKER@KSU.EDU.SA

Principal Investigator:

Samy Azer, Professor of Medical Education.

King Saud University, College of Medicine.

Medical Education Department.

Email: SAZER@KSU.EDU.SA

Dear Participants,

Thank you for taking the time to complete this questionnaire. We are Second-year medical students at King Saud University College of Medicine conducting a cross-sectional questionnaire-based study to assess the attitudes and perceptions of physicians from pediatric, family medicine, primary care physicians and residency trainees in these specialities (residents, fellows, consultants, and clinical academics) toward vaccine-hesitant parents.

Our study is based at King Saud University Medical City (KSUMC) and has been approved by the institutional review board.

Let me bring your attention, to the following:

- Participation is voluntary.
- All information obtained from the study will be kept confidential and used for medical research purposes only.
- All participants are anonymous.

By completing the questionnaire and signing the form, you are giving us permission to use the information in the research project, and possibly create a publication out of the work.

If you need additional information, you can contact the principal investigator at the email provided above.

Thank you again for your involvement in this study and your contribution.

Research Team:

Prof. S. Azer

MD. A. Albaker

Ms M. Alkhorayef

Ms N. Bin dakheel

Ms S. Almutairi

Ms S. Alhelal

Ms S. Maghrabi

| 1. Demographics |
| --- |

**Please select one answer for each of the following questions**

1. Age group:

- 25-34
- 35-44
- 45–54
- 55–64
- 64 and above

2. Sex:

- Female
- Male

3. Level of expertise:

- General practitioner
- Year 1 or 2 resident
- Year 3, 4 or 5 resident.
- Specialist
- Fellow
- Consultant
- Other

4. Field of practice:

- Primary care
- Paediatric Medicine
- Family Medicine

5. Years of practice:

- 10 years or fewer
- 11–20 years
- 21–30 years
- more than 30 years

6. Number of patients seen in a workday:

- 10 or fewer
- 11–15
- 16–20
- 21–25
- More than 25

7. Number of hours worked in a week:

- 30 or fewer
- 31–50
- more than 50

| 1. Experience with incompletely vaccinated Children |
| --- |

1. How frequently do you meet children who have missed one or more vaccines? Please select one option.

- Almost daily
- Weekly
- Monthly
- Few times a year

2. What are the reasons for the missed vaccines? (Choose all that apply)

- Child is ill
- Patient unable to reach the clinic due to work, family issues or emergencies, or lack of transportation
- Vaccines are contraindicated due to medical reasons such as immunodeficiency, chemotherapy
- Parents are hesitant because vaccines are painful
- Parents are hesitant because vaccines are not seen as beneficial
- Parents are hesitant because vaccines are harmful
- Other

| 1. Knowledge, beliefs, and opinions regarding vaccination |
| --- |

1. How confident are you in relation to vaccine related knowledge?

- Not confident at all
- Not very confident
- Somewhat confident
- Confident
- Extremely confident

2. How confident are you in relation to communication skills with vaccine-hesitant parents?

- Not confident at all
- Not very confident
- Somewhat confident
- Confident
- Extremely confident

**For the questions (Q1-10), please select one option to answer each question.**

***To what extent do you agree or disagree on the following statements:***

1. Childhood vaccines should be enforced by law such as no admission to school without complete childhood vaccination card.

- Strongly disagree
- Disagree
- Neutral
- Agree
- Strongly agree

1. Refusal of vaccinations by parents for nonmedical reasons should raise suspicions of negligence and questions about the child’s welfare.

- Strongly disagree
- Disagree
- Neutral
- Agree
- Strongly agree

1. Refusal of vaccinations by parents for nonmedical reasons should be dealt with as a child protection case and raised to the childhood welfare authority.

- Strongly disagree
- Disagree
- Neutral
- Agree
- Strongly agree

1. Refusing vaccinations is a parental right.

- Strongly disagree
- Disagree
- Neutral
- Agree
- Strongly agree

1. I feel angry when I deal with vaccine-hesitant parents.

- Strongly disagree
- Disagree
- Neutral
- Agree
- Strongly agree

1. I feel uncomfortable with internal conflict to continue the therapeutic relationship with parents refusing to vaccinate their kids.

- Strongly disagree
- Disagree
- Neutral
- Agree
- Strongly agree

1. I feel discomfort when I deal with vaccine-hesitant parents because they question my medical authority and appear mistrusting my professional judgement.

- Strongly disagree
- Disagree
- Neutral
- Agree
- Strongly agree

1. I feel that my role as a health advocate and medical expert is in danger because of parental vaccine rejection.

- Strongly disagree
- Disagree
- Neutral
- Agree
- Strongly agree

1. I feel indifference whether parents agree to vaccinate their children or not.

- Strongly disagree
- Disagree
- Neutral
- Agree
- Strongly agree

1. Dealing with vaccine-hesitant parents is outside my practice or responsibility.

- Strongly disagree
- Disagree
- Neutral
- Agree
- Strongly agree

| 1. Dealing with Vaccine-Hesitant Parents |
| --- |

**How frequently do you have appropriate discussions regarding vaccines with vaccine-hesitant parents?**

- Every encounter
- Frequently
- Sometimes
- Rarely

**For the next questions (Q1-6), please select one option to answer each question. *During vaccine discussions with vaccine-hesitant parents, how frequently do you discuss the following topics:***

1. Explore the reasons of vaccine refusal?

- Never
- Rarely
- Sometimes
- Frequently
- Always

2. Vaccine necessity

- Never
- Rarely
- Sometimes
- Frequently
- Always

3. Vaccine safety

- Never
- Rarely
- Sometimes
- Frequently
- Always

4. Catch-up schedules

- Never
- Rarely
- Sometimes
- Frequently
- Always

5. Vaccine efficacy

- Never
- Rarely
- Sometimes
- Frequently
- Always

6. MMR vaccine and autism concerns

- Never
- Rarely
- Sometimes
- Frequently
- Always

| 1. Response to refusal to vaccinate |
| --- |

**For the next questions (Q1-6), please select one option to answer each question.**

***How often do you:***

1. Require parents to sign a form to maintain record that the parents refuse vaccinations for non-medical reasons?

- Rarely
- Sometimes
- Frequently
- Always

2. Advise parents who have refused vaccination that they should inform on-call or urgent care physicians about their child's vaccination status?

- Rarely
- Sometimes
- Frequently
- Always

3. Dismiss families from your practice if they refuse one or more vaccines in the primary series?

- Rarely
- Sometimes
- Frequently
- Always

4. Schedule extra visits solely to address vaccination concerns?

- Rarely
- Sometimes
- Frequently
- Always

5. Advise parents who refuse certain vaccines that their child should wear MedicAlert tags or bracelets?

- Rarely
- Sometimes
- Frequently
- Always

6. Hold group information meetings or provide pamphlet to educate parents about vaccine related information?

- Rarely
- Sometimes
- Frequently
- Always

| 1. Challenges and strategies |
| --- |

**For the next questions (Q1-9), please indicate the extent to how far you find the following factors (relating to vaccine discussions) are challenging:**

1. Too many other issues to discuss during the clinic time

- Never been challenging
- Rarely challenging
- Sometimes challenging
- Always challenging

2. Lack of time allocated for vaccine talk

- Never been challenging
- Rarely challenging
- Sometimes challenging
- Always challenging

3. Concern that the talk would introduce conflict or hostility in the parent–physician relationship

- Never been challenging
- Rarely challenging
- Sometimes challenging
- Always challenging

4. Lack of knowledge about specific vaccines.

- Never been challenging
- Rarely challenging
- Sometimes challenging
- Always challenging

5. Lack of knowledge about current national vaccination schedule

- Never been challenging
- Rarely challenging
- Sometimes challenging
- Always challenging

6. Too many recent changes to the current national vaccination schedule, difficult to follow additions

- Never been challenging
- Rarely challenging
- Sometimes challenging
- Always challenging

7. Lack of knowledge about how vaccines work

- Never been challenging
- Rarely challenging
- Sometimes challenging
- Always challenging

8. Lack of knowledge about vaccine-preventable diseases

- Never been challenging
- Rarely challenging
- Sometimes challenging
- Always challenging

9. other reason: Please state ------------------------------------------------

**Which of the following would you like to be available to assist you having successful vaccine-related discussions? (Choose all that apply)**

- Pamphlets regarding vaccines that can be handed out to parents
- Websites and discussion forums
- Online training and course on vaccine related knowledge
- Face-to-face workshops
- Having a specialist clinic in each city to refer vaccine-hesitant parents who refuse one vaccine or more
- Having a hotline or a website available to parents to discuss vaccination
- Others…
